# Bayesian attenuation of offset analgesia filters out random disturbances in noxious stimuli

**DOI:** 10.1097/PR9.0000000000001359

**Published:** 2025-11-05

**Authors:** Ryota Ishikawa, Michihiro Osumi, Jun Izawa

**Affiliations:** a Ph.D. Program in Humanics, University of Tsukuba, Ibaraki, Japan; b Neurorehabilitation Research Center, Kio University, Nara, Japan; c Institute of Systems and Information Engineering, University of Tsukuba, Ibaraki, Japan

**Keywords:** Pain perception, Offset analgesia, Computational model, Bayesian integration, Noise filtering

## Abstract

**Introduction:**

Offset analgesia (OA), an endogenous pain inhibition after an abrupt decrease in noxious stimulation, provides a paradigm to study dynamic interaction between ascending and descending pain pathways. Previous studies assumed that this interaction follows deterministic dynamics. In contrast, a recent perspective views pain perception as a Bayesian process: a statistically optimal updating of pain predictions based on noisy sensory input.

**Objectives:**

We examined whether OA is driven by a deterministic interaction between ascending and descending pathways, or by a Bayesian process in which the brain updates pain perception by combining expectations with incoming signals.

**Methods:**

We modified the conventional OA paradigm by adding high-frequency noise after an abrupt decrease in noxious stimulation and measured pain intensity responses in healthy participants. Pain reports were analyzed using 2 computational models: a deterministic dynamic equation model and a recursive Bayesian integration model. Hypothesis testing was conducted using model selection.

**Results:**

Offset analgesia was observed after reduction of noxious stimuli, but pain was disinhibited by high-frequency disturbances. The deterministic model predicted unbounded oscillations depending on disturbance sequence, whereas the Bayesian model predicted gradual OA attenuation by filtering out noise. Model selection favored the Bayesian model.

**Conclusion:**

The brain dissociates noise from primary signals, achieving stable pain perception even in the presence of noisy inputs. Thus, OA reflects a stochastic integration between prediction and observation, with noise magnitude modulating pain intensity. Clinically, these results suggest that enhancing endogenous pain inhibition for chronic pain may be achieved through interventions targeting noise recognition mechanisms.

## Abstract 2

Supplemental Digital Content is Available in the Text.

Offset analgesia reflects stochastic integration processes of prediction and observation with noise filtering for robust pain perception, offering a revision of pain perception theories.

## 1. Introduction

Perception emerges from interactions of distributed and specialized neural responses among multiple brain regions.^4,10^ Pain perception, too, arises from interactions between ascending nociceptive signals from peripheral nerves and descending control signals from cortical regions, known as the descending pain modulatory system.^1,5,13,30,39,40^ These interactions are primarily mediated by the periaqueductal gray (PAG), which forms an inhibitory feedback circuit through a spinal–supraspinal–spinal loop.^41^ As a result, pain perception fluctuates over time in response to changes in nociceptive input. Offset analgesia (OA), a phasic attenuation of pain after an abrupt decrease in nociceptive stimulus,^16,42^ exemplifies this dynamic aspect of pain perception. To better understand the dynamic property, researchers have proposed a “dynamic equation model” that simulates the timing and shape of OA response,^6,32^ helping to predict how these neural interactions may produce transient changes in pain experience.

In contrast, our hypothesis is based on a modern perspective on the central nervous system that emphasizes its adaptability in the face of uncertainty.^2^ The brain maintains stable perception even when sensory inputs are inconsistent due to environmental changes.^34^ Recent theories of pain perception adopt a stochastic framework, such as Bayesian Brain Theory, which posits that the brain interprets pain by combining prior expectations with incoming sensory information.^7,17,35^ This process is thought to involve the prefrontal cortex, anterior cingulate cortex, and insular cortex, which also modulate the PAG.^35^ Accordingly, transient attenuation of pain triggered by an abrupt change in nociceptive input, as observed in OA response, may reflect the ongoing adjustment of pain prediction in response to sudden changes in noxious input. Importantly, this adjustment process remains effective even when noxious signals are noisy and unstable. Although recent studies have proposed that such real-time pain processing may follow Bayesian computation, empirical evidence remains limited.^33^

To distinguish between a previous model based on deterministic dynamic equations and a more recent model grounded in Bayesian theory, we modified the conventional OA paradigm by adding high-frequency disturbance signals (noise) after an abrupt decrease in noxious stimuli. The previous OA model, based on dynamic equations, explains offset analgesia as a result of slow oscillations triggered by an abrupt decrease in stimulus intensity. Because dynamical systems evolve deterministically, they respond directly to input signals, making pain perception highly sensitive to disturbance signals in the input sequence. This heightened sensitivity can induce excessive fluctuations, leading to unstable pain perception. In contrast, if pain perception operates through Bayesian inference by continuously updating prediction, high-frequency disturbance should be treated as noise and filtered out. Consequently, pain perception should remain stable regardless of disturbance sequence, while, on average, the overall pain intensity should be influenced by noise variance.

In this study, healthy participants reported pain intensity during an extended OA paradigm. Participant pain reports were analyzed using 2 computational models: 1 based on a previously published deterministic differential equation^6,32^ and the other on the newly formalized Bayesian model introduced here. The models were compared by assessing how well they fit the experimental data. The fundamental question is whether pain intensity becomes unstable in response to random noxious sequences or remains stable due to noise-filtering mechanisms. This research proposes a novel technique to assess individual pain perception processing by evaluating robustness against unpredictable noxious input, a challenge commonly encountered in daily life by both healthy individuals and patients.

## 2. Materials and methods

### 2.1. Participants

Twenty-two healthy volunteers (age: 21.8 ± 2.4, 9 females) participated in the main experiment. All participants gave informed consent before participating. The experiment was approved by the Institutional Review Board at the University of Tsukuba. All participants reported no history of neurological disorder and no tissue damage on the skin to be stimulated.

### 2.2. Thermal stimulation

**Figure 1.**
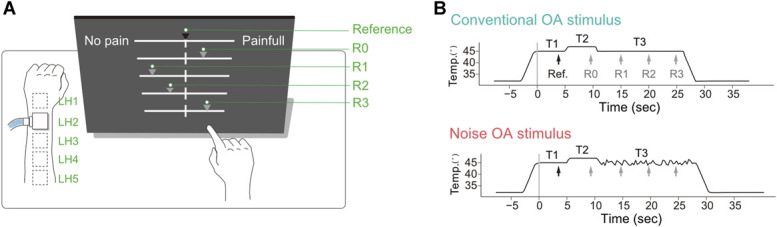
Experiment paradigm. (A) Experimental setup. The thermal stimulation was administered to a spot on the left or right arm (marked as dashed-line squares). The participant simultaneously rated the perceived pain using visual analogue scale (VAS) on the monitor with the nonstimulated hand. The black triangle on the top VAS denoted a pointer fixed at the center to remind the participant of the reference pain intensity on this scale. The other 4 gray triangles were operable pointers for rating responses at R0, R1, R2, and R3 timings. (B) Two thermal stimuli used to induce OA responses (top: conventional offset stimulus, bottom: offset stimulus with high-frequency signal). After the temperature reached the plateau (45°C), the stimulation consisted of T1, T2, and T3 phases. The arrows denoted the timings when the beep was presented. The first beep, the timing of which was denoted by a black arrow with “Ref.,” was presented for reference of pain. The following beeps, the timings of which were denoted by gray arrows with “R1, 2 or 3,” were presented for pain rating responses. OA, offset analgesia.

Thermal stimuli were provided with a thermosensory stimulator (TSA 2, Medoc, Ramat Yishai, Israel) to the skin via the thermode (CHEPS for TSA 2, 24 × 24 mm, Medoc). Stimulation positions were on the dorsal surfaces of hands (right hand: RH1 and left hand: LH1) and of forearms (right arm: RH2, RH3, RH4, and RH5; left arm: LH2, LH3, LH4, and LH5) (Fig. 1A). Ipsilateral positions were spatially separated by 5 cm and stimulated only once in the entire experiment so as to avoid peripheral habituation and sensitization. The order of site selection was consistent among participants; the right and left hands/arms were stimulated alternately from the distal to proximal sites (RH1, LH1, RH2, … RH5, and LH5).

#### 2.2.1. Conventional offset analgesia stimulus

To replicate a conventional offset analgesia phenomenon, the standard temporal pattern of thermal stimulus for the OA paradigm, ie, a conventional OA stimulus, was adapted from previous papers.^14,16,42^ At the initiation of each trial, the temperature remained at 32°C for 5 seconds. Afterward, it increased by 6°C/sec to the plateau (45°C). After reaching the plateau, there were 3 phases as the temperature changed incrementally. It first remained at 45°C for 5 seconds (T1 phase). Then, it increased to 47°C at 6°C/sec and remained for 5 seconds (T2 phase). Finally, it returned to 45°C and remained for 15 seconds (T3 phase) (Fig. 1B). After T3 phase terminated, the temperature decreased to 32°C at 6°C/sec.

#### 2.2.2. Noise offset analgesia stimulus

Stimuli including a high-frequency disturbance signal (noise) were the same as the conventional OA stimuli, except that during T3 phase, the temperature was the same 45°C on average, but fluctuated randomly at a frequency of 3 Hz (3 times per second) from 44°C to 46°C, at a change rate of about 10°C/sec. Two different temporal patterns of disturbance were tested (sequence #1 and #2).

These 2 OA stimuli were designed so that the mean temperature during the T3 phase was equivalent across stimuli. The applied temperature by the thermode were 44.99 ± 0.01°C (mean ± SD) for the conventional OA stimulus, 44.98 ± 0.78°C for noise OA stimulus (sequence #1), and 45.06 ± 0.79°C for noise OA stimulus (sequence #2). This small difference in average temperature (∼0.08°C difference at most) during the T3 phase is hard to influence participants' pain reports systematically.^20^

The noise amplitude (±1°C around 45°C) lies within the range where nociceptor responses are known to be approximately linear,^9,24^ and the change rate (∼10°C/s) is sufficient for detection by Aδ fibers.^31^ The pulse duration (ramps of ∼100 ms, holds of ∼200 ms) also falls within parameters reported to allow peripheral detection.^9^ Therefore, although peripheral attenuation at the skin–receptor interface is possible, the stimulus properties were sufficient for the fluctuations to be detected at the receptor level.

#### 2.2.3. Constant stimulus

Before experiencing the OA stimuli, participants completed a familiarization session in which the temperature remained at the plateau (45°C) throughout all phases. We refer to this as the “constant stimulus” condition.

### 2.3. Pain rating interface

Five visual analogue scales (VASs) for rating pain intensity were aligned vertically on the 21.5-inch touchscreen (Fig. 1A). The touchscreen was placed on a desk in front of the participant at eye level. The distance between the monitor and the participant's face was approximately 30 cm. The width of each VAS on the screen was 32.6 cm. The left edge of the VAS was labeled “No pain,” and the right edge was labeled “Painful” due to space constraints on the display. Before the task, participants were explicitly instructed that the right end of the VAS represented “the worst pain imaginable.”^14,16^ This instruction was provided verbally and emphasized to ensure consistent interpretation of the scale.

The top scale functioned as a fixed reference, with a dark gray pointer at the center (VAS = 50) indicating a pain rating in response to a plateau-level stimulus temperature (45°C). This method, modified from the previous work,^38^ offered 2 methodological advantages: (1) the fixed reference pointer allowed participants to report pain ratings consistently relative to the plateau-level stimulus, ensuring consistent reported responses across participants; (2) anchoring VAS = 50 at the plateau level for all participants, without performing temperature calibration for VAS = 50, kept the stimulus temperature within the same range (44–46°C) across participants, thereby allowing us to assume equivalent nociceptor activation levels among them.

The remaining 4 VASs, with movable pointers, corresponded to pain intensity at 4 consecutive time points within a single trial. The pointers were initially positioned at the center of the VASs. Participants used the index finger of their nonstimulated hand to indicate perceived pain on the touchscreen. All visual and interactive components of the rating interface were implemented using PsychoPy3.

### 2.4. Procedures

The participant sat in front of the monitor and placed the to-be-stimulated arm, dorsal side up, on the desk beside the monitor (Fig. 1A). In each trial, the experimenter attached the heat stimulator to one of the spots on the skin and initiated the stimulation. Five consecutive beeps were presented to signal event timing of the task. The first beep was presented 4 seconds after T1 phase started (Reference timing). The following beep was presented 4 seconds after T2 phase started (R0 timing), and the other beeps were presented 5 seconds, 10 seconds, and 15 seconds after T3 phase started (R1, R2, and R3 timing, respectively). These 4 beeps told the participant when to report pain intensity.

To become familiar with the plateau temperature and the pain rating interface, participants first performed 2 familiarization trials. To minimize the effect of repetition of the offset stimulus,^11^ they received only the constant stimulus where the temperature remained at a plateau level (45°C) throughout all phases.

In the subsequent 8 test trials, participants experienced 3 types of stimuli for offset analgesia: the conventional OA stimulus, the noise OA stimulus (sequence #1), and the noise OA stimulus (sequence #2). For each participant, the conventional OA stimulus was presented 4 times, whereas each noise OA stimulus (sequences #1 and #2) was presented twice. The first trial was always the conventional OA stimulus, followed by 7 trials consisting of the conventional OA stimulus and the 2 noise OA stimuli, presented in a randomized order. This predetermined randomized presentation sequence was not disclosed to the participants. All participants totally completed 2 familiarization trials and test trials with an intertrial interval of 3 minutes to avoid habituation or sensitization.

### 2.5. Model-naïve analysis

To test whether and how noise affects OA responses, we analyzed pain ratings on VAS measured during T3 phase only of trials presented with offset stimuli. First, to examine whether the OA stimulus followed by a high-frequency disturbance signal would lead to an OA response different than the conventional OA stimulus, we analyzed pain ratings under 2 factors: timing (R1, R2, and R3) and stimulus (conventional and high-frequency disturbance signals). We performed linear mixed-effect model (LMEM) analysis^3^ on participant pain ratings to evaluate the difference due to fixed effects (timing, stimulus, and interaction of these 2 factors) with “participant” as the random intercept. Here, timing factor was coded as R1 = −0.5, R2 = 0, and R3 = 0.5, whereas stimulus factor was coded as conventional = −0.5 and high-frequency signal = 0.5.

Furthermore, to examine effects of the sequence of random disturbances, we performed another LMEM analysis with timing (R1 = −0.5, R2 = 0, and R3 = 0.5) and sequence (sequence #1 = −0.5, sequence #2 = 0.5), and the interaction as fixed factors, with “participant” as the random intercept.

The random intercept accounted for the between-participant variability in the reported pain intensity and allowed us to test within-participant effects.^3^ We reported the effect size of each factor in LEME analyses (Cohen *d*)^21^ in addition to *P*-values. All data processing, statistical tests, and visualization were performed in R version 4.1.2 using the lme4,^3^ lmerTest,^23^ ggeffects,^25^ and EMAtools.^21^

### 2.6. Computational models and model-based analyses

Our main hypothesis, which proposes that pain perception follows a Bayesian process, and the alternative hypothesis based on deterministic dynamics,^6,32^ are each formalized using a set of equations in a straightforward manner (see Supplemental digital content, http://links.lww.com/PR9/A355 for details). We conducted computer simulations to predict the distinct time courses of pain intensity expected under each hypothesis. By comparing these simulated predictions with the experimental results, we determined which hypothesis was better supported by the data. To perform this analysis systematically, we used model fitting methods in which an optimization routine estimated the best parameters for each model to explain the experimental data. After adjusting the parameters, we evaluated how closely each model's predicted time course matched the observed data using information criteria, which reflects the goodness of fit. This approach to model-based analysis and hypothesis selection, which has been well established in research on motor control and decision making, is applied here to the study of pain perception.

## 3. Results

### 3.1. Model-naïve analyses

**Figure 2.**
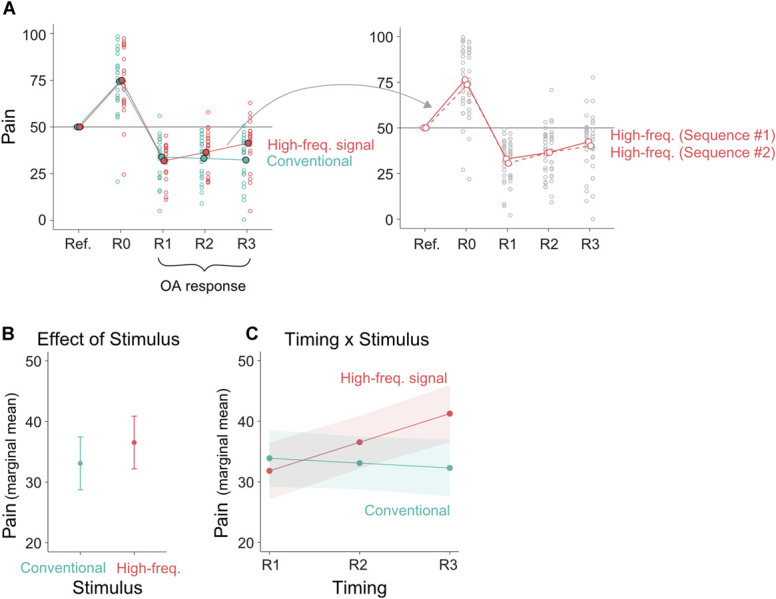
Model-naïve analysis. (A) Pain rating under the offset stimuli categorized by timing and stimulus factors (left) and pain categorized by timing and sequence factors (right). The unfilled circles represent the ratings averaged within individuals. The connected filled blue/red circles represent the average across individuals. Marginal mean of pain estimated by the LMEM: main effect of stimulus factor adjusting the other factors (B), interaction of timing and stimulus factors (C). The error bar or shading denote a 95% confidence interval. LMEM, linear mixed-effect model.

With respect to the plateau level (VAS = 50), pain intensity increased at R0 in response to the elevated temperature of the heat stimulation in T2 (Fig. 2A, left). Subsequently, it decreased beyond the reference at R1 in response to the temperature reduction to the baseline level in T3, demonstrating an analgesic effect. This attenuation persisted through R2 and R3, exhibiting a gradual recovery toward baseline. Thus, we successfully replicated the OA response.

The novel finding of this experiment is that the OA response profile, particularly during the spontaneous recovery phase, differed between the stimulus sequence without disturbance (conventional) and with high-frequency disturbance (high-freq.). This effect was confirmed by a significant positive effect of the stimulus factor (*P* = 1.10 × 10^−3^, Cohen *d* = 0.294, Table 1) (Fig. 2B). Although the OA effect gradually recovered toward baseline (timing factor: *P* = 2.19 × 10^−3^, Cohen *d* = 0.273), the rate of recovery was higher in the high-frequency disturbance than the conventional (timing × stimulus interaction: *P* = 1.79 × 10^−5^, Cohen *d* = 0.385) (Fig. 2C). Although we provided 2 disturbance sequences with similar frequency for high-frequency disturbance signals, we did not find a significant effect of the sequence itself (sequence factor: *P* = 0.343, Cohen *d* = −0.122, Table 2) (Fig. 2A, right). These results suggest that high-frequency signals after the offset, regardless of their sequence, consistently increased pain responses, ie, attenuated OA responses. This inseparable response to different disturbance sequences contradicts the deterministic hypothesis, which predicts a direct influence of the sequence on the response. Instead, our results support the stochastic hypothesis, in which the response is shaped by filtering out the disturbances, thereby reducing the influence of disturbance sequence differences on the response (see the simulation results below).

**Table 1:** Summary of the linear mixed-effect model analysis of timing and stimulus factors.

Factor	Estimate	SE	*t*-value	*P*	Cohen *d*
Intercept	34.18	2.15	16.15	1.10 × 10^−13^	—
Timing	3.92	1.27	3.07	**2.19** × **10**^**−**^**^3^**	0.273
Stimulus	3.44	1.04	3.30	**1.10** × **10**^**−**^**^3^**	0.294
Timing:Stimulus	11.04	2.54	4.33	**1.79** × **10**^**−**^**^5^**	0.385

Bold values indicate statistically significant effects (p < 0.05).

**Table 2:** Summary of the linear mixed-effect model analysis of timing and signal sequence factors.

Factor	Estimate	SE	*t*-value	*P*	Cohen *d*
Intercept	36.53	2.11	17.28	2.73 × 10^−14^	—
Timing	9.44	1.87	5.02	**9.70** × **10**^**−**^**^7^**	0.646
Sequence	−1.45	1.53	−0.94	0.343	−0.122
Timing:Sequence	0.09	3.75	0.02	0.979	0.003

Bold values indicate statistically significant effects (p < 0.05).

### 3.2. Model-based analyses

These alternative perspectives on pain perception mechanisms in the brain—deterministic and Bayesian—were embedded in the dynamic equation model and the recursive Bayesian integration model.

**Figure 3.**
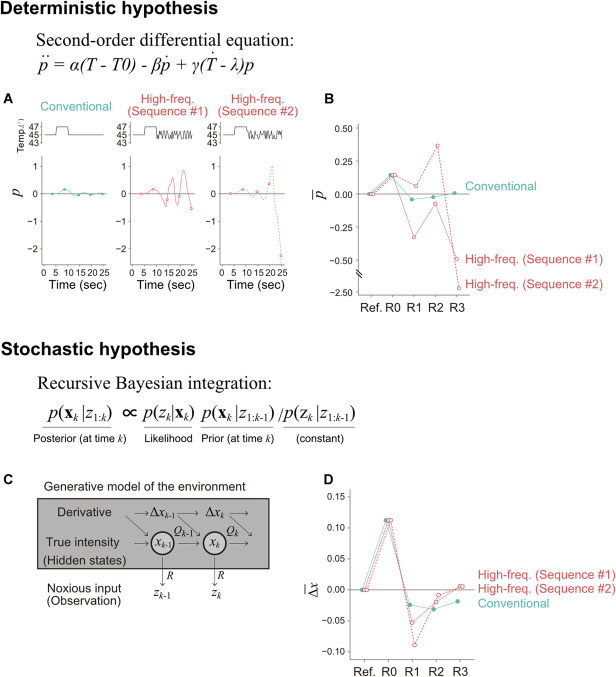
Schematic of the models and simulation results. (A) Model simulations under deterministic hypothesis using the second-order differential equation. The response (pain state, p) was simulated under each heat stimulation: control offset stimulus (left), offset stimuli after high-frequency signal with different sequences of sequence #1 and #2 (middle and right, respectively). (B) Pain state obtained by aggregating the simulated values within time periods corresponding to the response timings. (C) Model simulation under stochastic hypothesis using recursive Bayesian integration. The response ($∆x_{k})$ was simulated under each heat stimulation. (D) Phasic pain estimate obtained by aggregating the simulated values within time periods corresponding to the response timings.

To capture qualitative differences in model behavior in response to the abrupt offset change followed by the high-frequency disturbance signal, we simulated these 2 models. The deterministic dynamic equation model, as previously proposed,^6,32^ successfully replicated the OA response in the control condition (Fig. 3A, left) as a direct response of the differential equation to input signals. Consequently, it also responded differently to each disturbance sequence (Fig. 3A, sequence #1 and #2), predicting distinct pain intensities in the 2 sequences (Fig. 3B). However, this divergent response to high-frequency disturbances across different sequences contradicted participant consistent pain reports (Fig. 2A), contrary to the deterministic hypothesis.

In contrast, our recursive Bayesian integration model, in which pain intensity was predicted by the phasic component of pain state ($∆\bar{x}$) (Fig. 3C), successfully replicated the OA effect and the accelerated recovery driven by the high-frequency disturbance (Fig. 3D). Most importantly, the sequence did not significantly influence recovery profiles of OA. This is because the Bayesian model assumes that the brain treats high-frequency disturbance as noise. In this framework, the brain integrates prediction and observation while filtering out the disturbance sequence from its estimate of intensity (Fig. S1, http://links.lww.com/PR9/A355). With the high-frequency disturbance, the phasic pain estimate (a model of subjective pain perception)^8,18,44^ returned to the plateau-level more rapidly than in the conventional condition. This fast return under the high-frequency disturbance condition was independent of the noise signal sequence itself (sequence #1 or #2). These simulation results are consistent with the offset analgesia responses observed in the experiments.

These observations were quantitatively verified through comparison of the linear regression models based on information criteria (Tables S2-4, http://links.lww.com/PR9/A355), indicating that the phasic pain estimate model was preferred over the other models with strong or moderate evidence (*BF*_31_ = 1.27 × 10^135^, *BF*_32_ = 7.6) (Fig. S3, http://links.lww.com/PR9/A355).

## 4. Discussion

We found that OA was attenuated by the high-frequency disturbance signal superimposed on the abrupt change (offset) in the noxious stimulus. Furthermore, this attenuated OA profile occurred in either variation of disturbance sequences. These results contradict simulation results of the previous model of OA responses, which assumes that pain intensity follows a deterministic dynamic equation and that OA arises from the oscillatory behavior of this equation.^6,32^ This model predicted that pain intensity would diverge differently depending on the disturbance sequence in our extended OA paradigm. Thus, our results refute the deterministic perspective and rather provide empirical evidence that pain perception is mediated by a recursive Bayesian integration process.^18,33^ In the Bayesian process, the disturbance sequence added to the primary noxious signal is treated as noise and filtered out by assigning greater belief to the predicted pain than to the observed noxious inputs. Therefore, our findings suggest that real-time Bayesian processes are inherently embedded in human perception of pain.

This result is the first report that the high-frequency noise attenuates OA responses. To further confirm the reproducibility of this phenomenon, we ran a supplementary experiment by recruiting another 22 naïve, healthy volunteers (see Supplemental digital content, http://links.lww.com/PR9/A355 for details) and replicated them in a similar OA paradigm with a different sequence from the main experiment. We confirmed that the phasic pain estimate model was again preferred.

Our theory suggests that for stable pain perception, it is crucial to dissociate which sensations to believe or discard. However, the precise mechanism by which the brain accomplishes this dissociation remains unclear. Although prior studies have shown that OA is not disrupted by physiological manipulations, including experimental sensitization and opioid/opioid antagonist administration,^26,27^ in the present study, we manipulated the OA signal itself. By extending the current paradigm—such as by systematically varying the frequency of the signal—there is potential to reveal how the brain processes unpredictable noxious inputs to stabilize pain perception. The proposed experimental paradigm, which leverages random noxious disturbance, is capable of addressing this question by manipulating the quality of randomness.

Our results illuminate the computational basis of the interaction between the ascending and descending pathways of the pain perception mechanism.^1,5,13,30,39,40^ In the deterministic dynamic view, the descending inhibitory/excitatory signals from the PAG are the system's responses to ascending nociceptive signals; thus, the resultant perception is oscillatory. On the other hand, according to the perspective of Bayesian integration, the descending modulation via the PAG is determined by the level of pain prediction after filtering noise inherent in the ascending signals, which leads to stable perception and modulation of pain. In this process, the insula has a crucial role in computing pain prediction^19,36^ through Bayesian integration of prediction represented in prefrontal cortex and sensory inputs represented in the somatosensory regions. Since the insula is densely connected to the ventral striatum and amygdala,^15^ which respond to appetitive and aversive pain prediction,^12^ it is thought to control descending pain modulation via these subcortical regions to the PAG. Such control has to be online and recursive because the afferent nociceptive signal and the background noise change moment-to-moment. Thus, our results supporting the recursive Bayesian integration mechanism highlight the importance of stochastic inference computation embedded in the prefrontal cortex and insula for the pain modulation mechanism to the PAG.

Recent work by Onysk et al. (2024)^29^ also employed a Kalman filter framework to model pain processing; however, their approach estimates pain dynamics across discrete trials, whereas our model continuously tracks pain in real time. This distinction is critical for capturing transient within-trial phenomena such as offset analgesia, thereby underscoring the unique contribution of our real-time recursive Bayesian model to understanding dynamic pain modulation.

Our results also have clinical implications. Specifically, our theory suggests that attenuation of OA responses often reported in patients of chronic pain^22,37,43^ may be attributed to miscalculation of noise magnitude, eg, by experiencing large variability of perceptions due to tissue damage.^35^ Since the Bayesian integration process relies on knowledge of stimulus properties, eg, size of noise, enhancing participant learning about noxious stimulus properties should optimize regulation of spontaneous pain.^11^ In other words, the precision of encoding both extent and uncertainty in painful events^28^ is crucial to regulate pain perception. Thus, learning about noise levels in noxious stimuli, which might be also influenced by properties of the ascending pathway, may facilitate pain rehabilitation. Alternatively, neuromodulation of regions related to noise estimation, eg, insular, orbital frontal cortex, prefrontal cortex, or hippocampus,^36^ should be effective in enhancing this learning effect. Therefore, by utilizing noise-based regulation of pain-related learning, it may be possible to promote endogenous pain modulation and recovery from chronic pain.

## 5. Conclusion

We conclude that pain perception is robust against random disturbances in noxious signals, which are commonly present in daily life. To achieve this robustness, the brain continuously computes tonic and phasic pain predictions, adjusting them recursively based on the perceived magnitude of noise. Enhancing the ability to dissociate noise from true pain signals further strengthens stability of pain perception. Therefore, by leveraging this mechanism, enhancing Bayesian integration in the brain can facilitate rehabilitation for chronic pain.

## Disclosures

The authors declare that they have no competing interests.

## Supplemental digital content

Supplemental digital content associated with this article can be found online at http://links.lww.com/PR9/A355.
