# Supplementary material for: Bayesian attenuation of offset analgesia filters out random disturbances in noxious stimuli
Source: Pain Rep. 2025 Nov 5;10(6):e1359. doi: 10.1097/PR9.0000000000001359 (PMC12591703; doi:10.1097/PR9.0000000000001359)
Supplement: Supplementary file 1 [file painreports-10-e1359-s001.pdf]

Supplemental digital content (SDC) of

Bayesian attenuation of offset analgesia filters out random disturbances in noxious stimuli.

Author names and affiliations

Ryota Ishikawa[1], Michihiro Osumi[2], and *Jun Izawa[3]

[1]Ph.D. Program in Humanics, University of Tsukuba, Ibaraki 305-8573, Japan

[2]Neurorehabilitation Research Center, Kio University, Nara, Japan

[3]Institute of systems and Information Engineering, University of Tsukuba, Ibaraki 305-8573, Japan

*Corresponding author:

Jun Izawa

Faculty of Engineering, Information, and Systems

University of Tsukuba

1-1-1 Tennodai, Tsukuba, Ibaraki 305-8573, Japan

Tel.: +81-29-853-3756; Email: izawa@emp.tsukuba.ac.jp

1. Model-based analysis

1.1 Computational model for deterministic interactive dynamics hypothesis

The deterministic hypothesis posits that moment-to-moment pain perception is determined by the history of sensory input. Previous computational modeling formalized this concept as a second-order differential equation[1,5]:

$$\ddot{p} = \alpha(T - T_0) - \beta\dot{p} + \gamma(\dot{T} - \lambda)p, \qquad (S1)$$

where the temperature T and its first derivative $\dot{T}$ are both the inputs to the pain processing system. p represents "pain state", which determines pain intensity. $\dot{p}$ represents the first derivative, and $\ddot{p}$ represents the second derivative of the pain intensity.

For simulations, constant parameters were fixed according to estimated values in the previous study (α = 0.015, T_0 = 45, β = 0.325, γ = 0.07, and λ = 4.14)[1]. As inputs, we provided temperature patterns that were identical with those used in our experiment. Note that we ran simulations separately for the two variants of the signal sequence on offset stimuli (sequence #1 and #2) to see the difference in responses to these sequences. Since the simulation results of pain state (p) are continuous over time, we further assumed that participant reported pain intensity as an average over each time period ($\bar{p}$).

1.2 Computational model for stochastic inference hypothesis

(1) *Internal Model of pain stimulus generation*

We assume that the brain holds an internal model of the process by which noxious stimuli are generated in the environment[2]. The extent and transition of pain event are represented by a current noxious signal x_k and its change Δx_k, where k is time step ($\Delta t_{k:k-1} \equiv 1/3$ sec. in this paper). Thus, evolution and volatility of a noxious state are described by a difference equation of $\mathbf{x}_k = [x_k \quad \Delta x_k]^T$ with

$$\mathbf{x}_{k+1} = A\mathbf{x}_k + \mathbf{w}_k, \qquad \mathbf{w}_k \sim \mathcal{N}(0, Q_k), \qquad (S2)$$

where $\mathbf{w}_k$ is time-varying environmental noise with a covariance matrix $Q_k = \begin{bmatrix} q_k & 0 \\ 0 & q_k \end{bmatrix}$. The transition matrix A is defined as $\begin{bmatrix} 1 & 1 \\ 0 & 1 \end{bmatrix}$. In contrast to the deterministic model,[1] we assume that the brain does not sense the change of noxious stimuli Δx_k ($\dot{T}$ in the deterministic model[1]) directly, but it observes the current noxious signal x_k via peripheral nerves influenced by sensory noises:

$$z_k = x_k + v = H\mathbf{x}_k + v, \qquad v \sim \mathcal{N}(0, R) \qquad (S3)$$

where v is an observation noise defined by a time-constant variance R. The observation matrix H has a structure of $[1 \quad 0]$. Without loss of generality, z_k was coded relative to the baseline temperature, 45°C. Thus, at $k = 1$, the state is set as $\mathbf{x}_1 = [0 \quad 0]^T$ because the initial stimulus intensity of each trial was always presented as 45°C (**Fig. 1b**).

Under the control condition, the magnitude of the system noise was set $q_k = q_0$ (default) in all time steps, while in the disturbance signal condition it was set to $q_k = q_0$ except during the T3 phase with additional noise, $q_k = q_0 + \Delta q$.

(2) *Recursive Bayesian Integration of predictions and observations.*

When the posterior probability distribution of the pain state at $k-1$ is represented by $p(\boldsymbol{x}_{k-1}|z_{1:k-1})$, the predictive prior probability at k is given by $p(\boldsymbol{x}_k|z_{1:k-1}) = \int p(\boldsymbol{x}_k|\, \boldsymbol{x}_{k-1})p(\boldsymbol{x}_{k-1}|z_{1:k-1})d\boldsymbol{x}_{k-1}$ in which the transition probability $p(\boldsymbol{x}_k|\boldsymbol{x}_{k-1})$ follows Eqn. (S2). The Bayesian integration updates $p(\boldsymbol{x}_k|z_{1:k-1})$ after observing a new data point z_k by $p(\boldsymbol{x}_k|z_{1:k}) = p(z_k|\boldsymbol{x}_k)p(\boldsymbol{x}_k|z_{1:k-1})/p(z_k|z_{1:k-1})$, where the likelihood function $p(z_k|\boldsymbol{x}_k)$ follows Eqn. (S3). In the case of linear Gaussian system, this recursive Bayesian can be represented with linear update equations separating the mean and the variance of the probability distribution, known as Kalman filtering[3]. In this framework, the predicted state estimate is $\hat{\mathbf{x}}_{k|k-1} = A\hat{\mathbf{x}}_{k-1|k-1}$and its uncertainty covariance is $P_{k|k-1} = AP_{k-1|k-1}A^T + Q_k$. Then, their update after the new observation is

$$\hat{\mathbf{x}}_{k|k} = \hat{\mathbf{x}}_{k|k-1} + K_k\left(z_k - H\hat{\mathbf{x}}_{k|k-1}\right), \qquad (S4)$$

and $P_{k|k} = (I - K_kH)P_{k|k-1}$, where $K_k = P_{k|k-1}H^T(H_kP_{k|k-1}H^T + R_k)^{-1}$.

For the linear fitting below, we simulated this Kalman filter model to generate time series data of $\hat{x}_k$ and $\Delta\hat{x}_k$, then, took their averages within the corresponding time periods ($\bar{x}$ and $\Delta\bar{x}$) to obtain regressors at response timings in the task: R0, R1, R2, and R3.

Free parameters are (q_0), additional system noise (Δq) in the T3 phase, and observation noise (R). In a Kalman filtering framework, the ration between the system noise and the measurement noise determines the update rates. Thus, without lack of generality, we set R = 1 and explored the optimal q_0 and Δq to explain the data. To this end, we tested all pairs of q_0 and Δq, within a range wherein Kalma gain[3] equals $0.5\times[10^{-10}, 10^{-9}, \ldots, 10^{0}]$, totaling 11×11 combinations of the parameters.

1.3 Linear fitting and model comparison

To examine which model and state variables best explain pain intensity, we conducted a linear mixed-effects model analysis and compared the models using Bayesian Information Criteria (*BIC*)(**Tables S2–4**).

The first model is the "Deterministic interactive dynamics model" (Eqn. (S1)) that predicts the perceived pain intensity by using the pain state $\bar{p}_{(t,s)}$ as a regressor for each subject[1]. This model is described using pseudo-R notation as follows

$$Pain_{(t,s,i)} \sim \beta_0 + b_i + \beta_1 \bar{p}_{(t,s)}, \qquad (S5)$$

where *Pain* is the participant's report of pain intensity, t is the timing index (R0, R1, R2, and R3), s is the stimulus index (control, disturbance sequence #1, and disturbance sequence #2), and i is the individual index. β_0 is the fixed intercept, b_i is the random intercept, and β_1 is the coefficient of the pain state.

The second model is the "current pain intensity model". This model is based on the stochastic integration model (Eqn. (S2) and (S3)) and uses the estimate of the current pain state $\bar{x}_{(t,s)}$ as a regressor:

$$Pain_{(t,s,i)} \sim \beta_0 + b_i + \beta_1 \bar{x}_{(t,s)}. \qquad (S6)$$

The third was the "phasic pain estimate model". This model is also based on the stochastic integration model (Eqn. (S2) and (S3), but uses the change of the pain state $\Delta\bar{x}_{(t,s)}$ as a regressor.

$$Pain_{(t,s,i)} \sim \beta_0 + b_i + \beta_1 \Delta\bar{x}_{(t,s)}. \qquad (S7)$$

Model comparison was performed based on *BIC* calculated from each linear model (Eqn. (S5), (S6), and (S7)). A lower value of *BIC* indicates better fit. Further, we computed the Bayes factor (*BF*). In general, *BF* of Model 1 over Model 2 is approximated from a *BIC* difference between the models: $BF_{12} \cong \exp\left(\frac{BIC_2 - BIC_1}{2}\right)$, where BIC_1 and BIC_2 denotes the *BIC* values for Models 1 and 2, respectively.[6] $BF_{12} = 1$ indicates that both models are equally likely. Within the interval 1/3 to 3, it indicates only anecdotal evidence. $BF_{12} > 3$ or $BF_{12} < 1/3$ indicates moderate evidence in favor of Model 1 or Model 2, respectively. $BF_{12} > 10$ or $BF_{12} < 1/10$ indicates strong evidence.[4]

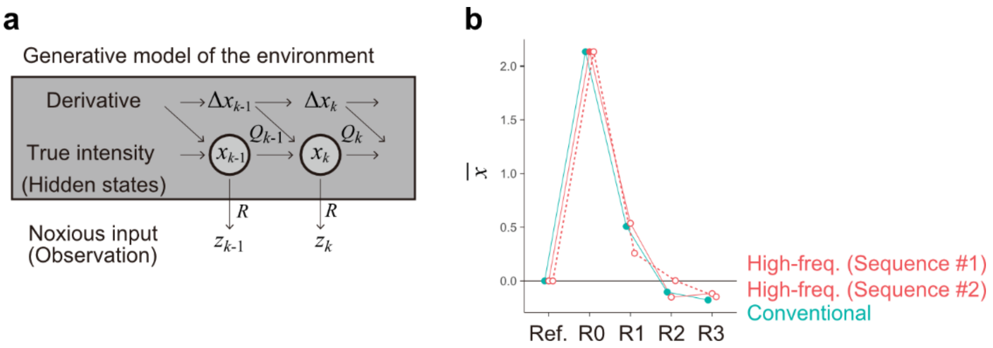

Figure S1. Model simulation results. (a) Generative model for the stochastic integration hypothesis. **(b)** Model simulation results of the current pain estimate, categorized by stimulus condition.

Table S1. LMEM analysis of the deterministic interactive dynamics model.

Factor	Coefficient	SE	*t*-value	*p*-value	Cohen' *d*
Intercept	45.59	2.00	22.76	$< 2\times10^{-16}$	-
**Pain state**	5.67	1.44	3.91	$\mathbf{9.92\times10^{-5}}$	0.29

Table S2. LMEM analysis of the stochastic inference model regarding the current pain estimate

Factor	Coefficient	SE	*t*-value	*p*-value	Cohen' *d*
Intercept	34.26	2.01	16.97	1.42×10^{-14}	-
**Current Pain Estimate**	17.51	0.54	32.31	$< \mathbf{2\times10^{-16}}$	2.47

Table S3. LMEM analysis of the stochastic inference model regarding the phasic pain estimate

Factor	Coefficient	SE	*t*-value	*p*-value	Cohen' *d*
Intercept	42.59	1.99	21.37	3.14×10^{-16}	-
**Phasic Pain Estimate**	278.17	8.56	32.47	$< \mathbf{2\times10^{-16}}$	2.48

2. Supplementary experiment

2.1 Methods

The aim of the supplementary experiment was to reproduce the effect of a high-frequency disturbance signal in a different task sequence. Twenty-two healthy naïve participants were recruited for the supplementary experiment (age: 20.8±1.7, twelve females). The experimental design was the same as that of the main experiment, except that half of the eight test trials were presented with a stimulus that does not induce OA responses, i.e., the temperature decreased, rather than increasing, in the T2 phase. Two additional test trials were presented with a control offset stimulus and the other two trials were presented with the same offset stimuli as the main experiment, which were also followed by the same high-frequency disturbance signals.

2.2 Results

2.2.1 Model-naïve analysis

Since we sought to confirm the effect of the high-frequency disturbance signal for OA responses, we analyzed only trials presented with OA stimuli (**Fig. S2a**). Thus, the design of LMEM analyses was the same as that of the main experiment.

We replicated the effect of high-frequency disturbance signal observed in the main experiment as we found a significant positive effect of the Stimulus factor ($p = 3.44\times10^{-4}$, Cohen's $d = 0.442$) (**Fig. S2b** and **Table S4**). Also, the effect of the Timing factor was significant ($p = 3.35\times10^{-2}$, Cohen's $d = 0.274$), but the interaction was not (Timing × Stimulus: $p = .621$, Cohen's $d = 0.063$). Moreover, the signal affected the OA response irrespective of sequence (Sequence factor: $p = 0.506$, Cohen's $d = 0.127$, **Table S5**). These results demonstrated that the high-frequency disturbance signal following the offset robustly attenuated OA responses, which was independent of the signal sequence. Thus, we reproduced the effect of the high-frequency disturbance on OA effect.

2.2.2 Model-based analysis

We fitted the models to all data. We reproduced the strong evidence supporting the phasic pain estimate model over the current pain state model ($BF_{32} = 12.83$) and the deterministic dynamical interaction model ($BF_{31} = 2.48\times10^{77}$).

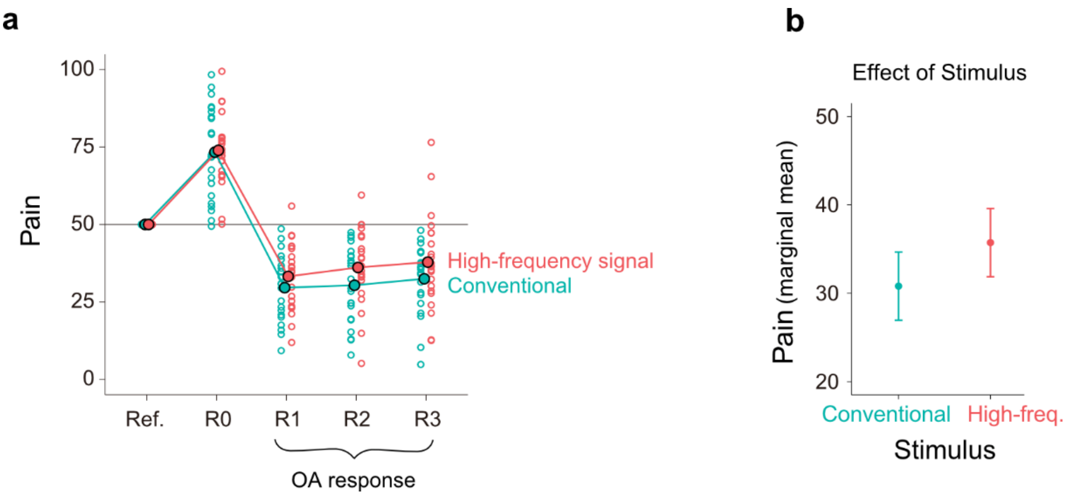

Figure S2. Model-naïve analysis of Supplementary Experiment. (a) Pain under the offset and onset stimuli categorized by Timing factor per stimulus. Unfilled circles represent ratings averaged within individuals. Connected filled blue/red circles represent averages across individuals. **(b)** Marginal mean of pain estimated by the LMEM: main effect of Stimulus factor adjusting other factors. The error bar represents a 95% confidence interval.

Table S4. Summary of the LMEM analysis on timing and stimulus factors (Supplementary Experiment).

Factor	Estimate	SE	*t*-value	*p*-value	Cohen' *d*
Intercept	33.26	1.83	18.13	1.02×10^{-14}	-
**Timing**	3.74	1.75	2.13	$\mathbf{3.35 \times 10^{-2}}$	0.274
**Stimulus**	4.92	1.42	3.44	$\mathbf{3.44 \times 10^{-4}}$	0.442
Timing:Stimulus	1.73	3.50	0.49	.621	0.063

Table S5. Summary of the LMEM analysis on timing and signal sequence factors (Supplementary Experiment).

Factor	Estimate	SE	*t*-value	*p*-value	Cohen' *d*
Intercept	35.73	2.12	16.81	4.86×10^{-14}	-
Timing	4.60	2.55	1.80	.074	0.343
Sequence	1.39	2.08	0.66	.506	0.127
Timing:Sequence	-1.37	5.11	-0.26	.788	-0.051

3. Robustness of model comparison results

To examine whether the observed superiority of the recursive Bayesian integration model depends on the choice of parameters in the dynamical systems model, we conducted simulations exploring a broad range of parameter settings.

In the analyses of the main manuscript, simulations used the median parameter set estimated from 11 subjects reported by Cecchi et al. (2012)[1]. To test the robustness of our conclusion beyond this empirically supported range, we generated 100 parameter sets by sampling each parameter randomly from a uniform distribution bounded between 0.01 times the minimum and 100 times the maximum values reported by Cecchi et al. This approach enabled a comprehensive exploration in the conceivable parameter space, which is beyond even a plausible range according to the previous report [1]. For each parameter set, we computed the Bayes Factor comparing the recursive Bayesian integration model and the dynamical systems model. As illustrated in **Fig. S3**, the Bayesian model consistently showed a stronger explanation ability than the dynamical system model, across all tested parameterizations.

These results demonstrate the generality of the superiority of the recursive Bayesian integration model across a conceivable range of parameter configurations.

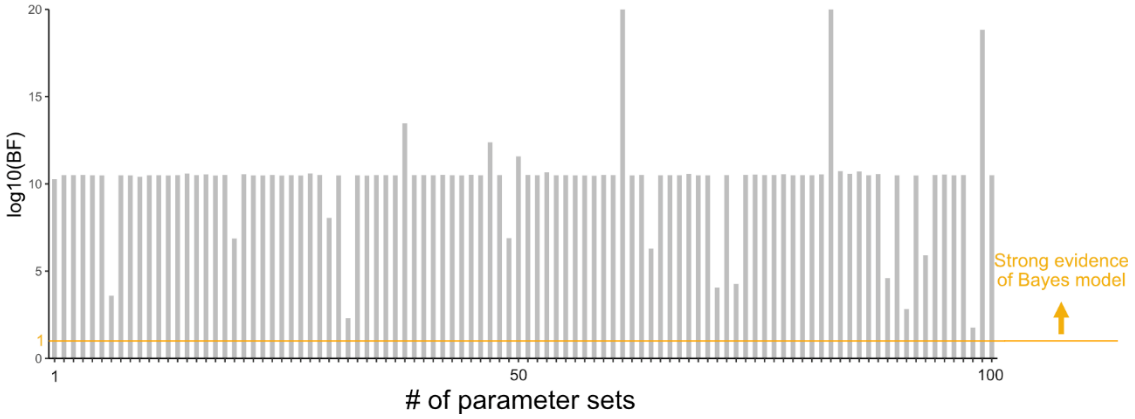

Figure S3. Bayes Factors (log scale) comparing the recursive Bayesian integration model to the dynamical systems model across 100 randomly sampled parameter sets. Parameters were sampled uniformly within a range from 0.01 times the minimum to 100 times the maximum values reported by Cecchi et al. (2012). In all cases, the Bayes Factors exceed 10 ($\log_{10}(BF) > 1$), indicating strong evidence favoring the Bayesian model over the dynamical systems model across a broad parameter space. This result underscores the robustness of our main findings.

References

[1] Cecchi GA, Huang L, Hashmi JA, Baliki M, Centeno MV, Rish I, Apkarian AV. Predictive dynamics of human pain perception. PLoS Comput Biol 2012;8:e1002719.

[2] Jepma M, Koban L, van Doorn J, Jones M, Wager TD. Behavioural and neural evidence for self-reinforcing expectancy effects on pain. Nat Hum Behav 2018;2:838–855.

[3] Kalman RE. A new approach to linear filtering and prediction problems. J Basic Eng 1960;82:35–45.

[4] Kass RE, Raftery AE. Bayes Factors. J Am Stat Assoc 1995;90:773–795.

[5] Petre B, Tetreault P, Mathur VA, Schurgin MW, Chiao JY, Huang L, Apkarian AV. A central mechanism enhances pain perception of noxious thermal stimulus changes. Sci Rep 2017;7:3894.

[6] Wagenmakers E-J. A practical solution to the pervasive problems of p values. Psychon Bull Rev 2007;14:779–804.
